# Mesenchymal stromal cells for treatment of steroid-refractory GvHD: a review of the literature and two pediatric cases

**DOI:** 10.1186/1755-7682-4-27

**Published:** 2011-08-15

**Authors:** Caroline M Wernicke, Thomas GP Grunewald, Juenger Hendrik, Selim Kuci, Zyrafete Kuci, Ulrike Koehl, Ingo Mueller, Michaela Doering, Christina Peters, Anita Lawitschka, Hans-Jochem Kolb, Peter Bader, Stefan Burdach, Irene von Luettichau

**Affiliations:** 1 Children's Cancer Research and Roman Herzog Comprehensive Cancer Center, Department of Pediatrics, Klinikum rechts der Isar, Technische Universität München, Kölner Platz 1, 80804 Munich, Germany; 2 Medical Life Science and Technology Center, TUM Graduate School, Technische Universität München, Boltzmannstrasse 17, 85748 Garching, Germany; 3 Division for Stem Cell Transplantation, Department of Hematology, Oncology and Hemostasis, Hospital for Children and Adolescents, University of Frankfurt, Theodor-Stern-Kai 7, 60590 Frankfurt am Main, Germany; 4 University Medical Center Hamburg-Eppendorf, Martinistrasse 52, 20246 Hamburg, Germany; 5 University Children's Hospital, Hoppe-Seyler-Strasse 1, 72076 Tuebingen, Germany; 6 St. Anna Children's Hospital, Kinderspitalgasse 6, 1090 Vienna, Austria; 7 Division for Stem Cell Transplantation, Department of Medicine III, Klinikum rechts der Isar, Technische Universität München, Ismaninger Strasse 22, 81675 Munich, Germany

## Abstract

Severe acute graft versus host disease (GvHD) is a life-threatening complication after allogeneic hematopoietic stem cell transplantation. Human mesenchymal stromal cells (MSCs) play an important role in endogenous tissue repair and possess strong immune-modulatory properties making them a promising tool for the treatment of steroid-refractory GvHD. To date, a few reports exist on the use of MSCs in treatment of GvHD in children indicating that children tend to respond better than adults, albeit with heterogeneous results.

We here present a review of the literature and the clinical course of two instructive pediatric patients with acute steroid-refractory GvHD after haploidentical stem cell transplantation, which exemplify the beneficial effects of third-party transplanted MSCs in treatment of acute steroid-refractory GvHD. Moreover, we provide a meta-analysis of clinical studies addressing the outcome of patients with steroid-refractory GvHD and treatment with MSCs in adults and in children (n = 183; 122 adults, 61 children). Our meta-analysis demonstrates that the overall response-rate is high (73.8%) and confirms, for the first time, that children indeed respond better to treatment of GvHD with MSCs than adults (complete response 57.4% vs. 45.1%, respectively).

These data emphasize the significance of this therapeutic approach especially in children and indicate that future prospective studies are needed to assess the reasons for the observed differential response-rates in pediatric and adult patients.

## Background

Allogeneic stem cell transplantation (SCT) is a potentially curative treatment option for different hematologic disorders and is increasingly included into therapy protocols for solid tumors due to the potential immunologic effect of donor T-cells on minimal residual disease [1]. Steroid-refractory acute and chronic graft versus host disease (GvHD), however, remain a therapeutic challenge and are associated with high mortality rates and poor quality of life in surviving patients [2,3]. A novel promising approach for the treatment of steroid-refractory GvHD is the application of human mesenchymal stromal cells (MSCs) [4,5].

These multipotent non-hematopoietic progenitor cells are found in the bone marrow but also in many other tissues [6,7]. They can be identified by their phenotypic and functional characteristics, exhibit high multi-lineage plasticity and can differentiate into adipocytes, chondrocytes and osteoblasts [6,7]. Moreover, they possess self-renewal capacity and thus seem to play an important role in endogenous tissue regeneration [6,7]. Of note, MSCs also have immune-modulatory features and can promote peripheral tolerance, i.e. by inhibiting T- and B-cell proliferation [6,8]. These features suggest that MSCs may represent an innovative therapeutic tool in immune-mediated disorders such as GvHD as reviewed below.

### Biological properties of MSCs

MSCs were first described by Friedenstein et al. in 1974 [9]. They can be isolated from various tissues such as bone marrow, peripheral blood, adipose tissue and placenta [7,10]. MSCs have large self-renewal capacity *in vitro *while maintaining their multipotency [7,10]. Hence, they can give rise to several distinct mesenchymal tissues, i.e. bone, cartilage, tendon, muscle, and fat [7,10]. Accordingly, they are believed to have an important role in tissue repair [7,10]. Furthermore, MSCs have a wide range of suppressive effects on cells of the adaptive and innate immune system [11,12]. They suppress CD4^+ ^and CD8^+ ^T-lymphocyte proliferation and modulate their functional response leading to a decrease of interferon γ (IFNγ), interleukin 2 and tumor necrosis factor α (TNFα) production, but to an increase of interleukin 4 secretion [13]. Moreover, MSCs can induce anti-inflammatory regulatory T-cells (T-regs) [14] that ultimately may attenuate T-cell cytotoxicity [15]. Besides their effects on T-cells, MSCs also suppress B-cell differentiation and proliferation [16,17]. In addition, activated MSCs can block the maturation of dendritic cells [18], which are essential in induction of immunity and tolerance, and have been shown to suppress natural killer cell proliferation and cytotoxicity [19]. These immunosuppressive functions of MSCs seem to require preliminary activation by immune cells themselves through the proinflammatory cytokine IFNγ alone or in combination with TNFα, interleukin 1α or 1β [20,21], which points to an auto-regulatory feedback loop of MSCs and immune cells at sites of tissue inflammation. To date, it is not fully understood how MSCs exert their immuno-regulative functions, but they seem to be mediated by the cumulative action of several soluble factors such as indoleamine-2,3-dioxygenase [22,23], prostaglandin E2 [13,24,25], and interleukin 6 [12,26], all of which are secreted by MSCs upon activation. Endogenous MSCs can be activated and mobilized if needed, e.g. for tissue repair [27]. However, the efficiency is very low, possibly explaining why for example damaged muscles heal very slowly [27]. After intravenous application, most MSCs home into lymphoid organs directed at least partially by chemokine receptors and their ligands [28]. Thus, it appears that in a preclinical setting, a direct injection or placement of MSCs into the damaged site in need for repair may be superior to vascular delivery [28,29]. In addition, vascular delivery may suffer from a "pulmonary first pass effect" whereby intravenously injected MSCs are sequestered in the lungs [29]. However, intravenous application may still be advantageous in some instances, because MSCs will be subjected to signals within the circulation on their way to the adequate lesion, thus mimicking the physiological situation. In accordance, MSCs are recruited into the area of inflammatory bowel disease and facilitate mucosal repair in an experimental mouse model [30]. At present, MSCs are under preclinical investigation or are already employed as new therapeutics in tissue repair and the treatment of otherwise refractory auto-immune diseases such as systemic lupus erythematosus as well as transplantation-associated acute and chronic GvHD, as reviewed below [6,7,31-34].

### MSCs in tissue repair

For the clinical purpose of tissue repair, MSCs have been most widely used for their potential in orthopedic applications [35-37], skin lesions [38,39] and in treatment of cardiovascular diseases [7,10,40-43]. For instance, Wakitani et al. reported on the successful transplantation of autologous cell-culture expanded MSCs into nine full-thickness articular cartilage defects of the patello-femoral joints of three patients [37]. Six months after transplantation, the patients' clinical symptoms had improved and the improvements have been maintained over the follow-up period of about two years indicating feasibility and safety of this approach [37]. Consistently, in a consecutive long-term follow-up study of 41 patients Wakitani and colleagues did not record any adverse-effects of this method including tumorigenesis and infections [35]. Moreover, a recent pilot-study demonstrated the replenishment of type VII collagen and re-epithelialization of chronically ulcerated skin after intradermal administration of allogeneic MSCs in two patients with recessive epidermiolysis bullosa - a severe inherited skin-blistering disorder caused by mutations in the *COL7A1 *(collagen, type VII, alpha 1) gene [38]. Similarly, Wagner et al. reported on the treatment of six patients with recessive epidermiolysis bullosa with allogeneic bone marrow transplantation [39]. All patients showed improved wound healing and a reduction in blister formation possibly suggesting that bone marrow-derived MSCs might have contributed to skin repair [39]. In analogy, as there is compelling preclinical evidence for safety and efficacy of this approach in animal models, there are to date several ongoing clinical trials studying the role of MSCs in therapy of cardiovascular diseases including myocardial infarction and hypertrophy (for review see [44]). In these trials, also the most efficient mode of application shall be assessed (e.g. direct myocardial, systemic and/or intracoronary injection) [44].

### MSCs in treatment of auto-immune diseases

Due to their immuno-suppressive properties, MSCs are currently tested for their use in autoimmune diseases such as multiple sclerosis and Crohn's disease as well as systemic lupus erythematodes, systemic sclerosis and type 1 diabetes mellitus [45-47]. The first disease in which the therapeutic potential of MSCs was addressed was in a murine model of multiple sclerosis [47]. Here, intravenous administration of syngeneic MSCs resulted in clinical and histological improvement, which correlated with time of MSCs treatment (the earlier the better) [47]. These promising data were confirmed by other groups [46,48,49] and supported by the finding that autologous bone marrow-derived MSCs can exert anti-proliferative effects on T-cells from healthy donors and those from patients with autoimmune diseases like rheumatoid arthritis, systemic lupus erythematodes and Sjoegren's syndrome [50]. Moreover, MSCs injection into diabetic mice caused the disappearance of β-cell-specific T-cells from diabetic pancreas suggesting that MSCs might be a possible option also for treatment of auto-immune diabetes mellitus [51]. In summary, these preclinical results underscore the concept of autologous MSCs for treatment of patients with autoimmune diseases, which now has to be validated in clinical trials.

### MSCs in treatment of GvHD

To date, MSCs have been safely administered for treatment of severe steroid-refractory GvHD in adults [4,52-60] and, to a far lesser extent, in children [61-63]. In a landmark study, Le Blanc et al. reported on the successful treatment of severe steroid-resistant grade IV GvHD of the gut and the liver after unrelated allogeneic SCT in a 9-year-old boy with haploidentical third-party bone marrow-derived MSCs [64]. This observation was supported by a multicenter non-randomized phase II study addressing the infusion of MSCs from either HLA-identical stem cell donors, haploidentical family donors or unrelated HLA-mismatched donors in 55 patients with severe steroid-refractory GvHD [4]. 30 out of 55 patients had a complete response and 9 patients showed improvement of GvHD, indicating that, irrespective of the donor, MSCs might be an effective therapy for patients with steroid-resistant acute GvHD [4]. Interestingly, children tended to respond consistently better than adults, with more complete remissions and less progressive disease (response-rate approximately 80% in children compared with 60% in adults) [4]. This finding is further substantiated by our meta-analysis addressing the differential outcome of adults and children with steroid-refractory GvHD treated with MSCs (see also Table 1 and Table 2). In another landmark study, Lazarus et al. hypothesized that cotransplantation of MSCs and hematopoietic stem cells (HSCs) from human leukocyte antigen (HLA)-identical sibling donors after myeloablative therapy could facilitate engraftment and ameliorate GvHD [55]. Their open-label, multicenter trial addressing MSCs together with HSCs to 46 patients with hematologic malignancies showed safety and feasibility of this approach [55]. Consistently, Muller et al. reported on the use of MSCs in treatment of GvHD in 7 pediatric patients after allogeneic SCT with a maximum follow-up of 29 months and did not observe adverse effects, but stabilization of graft function and improvement of GvHD [65]. Moreover, preliminary data reported in abstract form of a company-sponsored randomized, placebo-controlled multicenter phase III trial for steroid-resistant severe acute GvHD addressing third-party MSCs (Prochymal^®^) to 163 patients and placebo to 81 patients showed improved complete and partial response-rates in patients with gut and liver involvement (82% vs. 68% and 76% vs. 47%, respectively) [66]. Taken together, these studies suggest that third-party transplanted MSCs are at least a feasible treatment option for otherwise steroid-refractory GvHD in adult as well as pediatric patients. However, data on MSCs efficacy in treatment of GvHD have to be considered with caution. For instance, although the aforementioned placebo-controlled multicenter phase III study showed a statistical superiority of MSCs over placebo in patients with gut and liver GvHD [66], it remains unclear why MSCs showed no improvement in patients with skin GvHD [67]. The disparate results between this study and other studies mentioned above may be in part explained by the great heterogeneity of production and processing of MSCs in different reports (e.g. source, age of donors, culture conditions, number of passages etc.) [67]. Hence, it is difficult to draw definitive conclusions on MSCs efficacy in treatment of GvHD and a consensus on a common protocol may be useful to overcome this obstacle.

**Table 1:** Summary of clinical studies addressing the outcome of patients with steroid-refractory GvHD treated with MSCs

				outcome (total)			outcome (%)			
study	# of patients (children/adults)	mean age (years)	sex (m/f)	CR	PR	NR	CR	PR	NR	reference
1	19 adults	27.5	14/5	4	10	5	21.1	52.6	26.3	Weng JY 2010
2	7 children	14	ns	3	1	3	42.9	14.3	42.9	Muller I 2008
3	55 (25 children, 30 adults)	22	34/21	30	9	16	54.5	16.4	29.1	Le Blanc K 2008
4	12 adults	ns	ns	3	6	3	25.0	50.0	25.0	Zhang LS 2009
5	13 adults	58	7/6	1	1	11	7.7	7.7	84.6	von Bonin M 2009
6	2 children, 6 adults	43.25	7/1	6	0	2	75.0	0.0	25.0	Ringdén O 2006
7	31 adults	52	21/10	24	5	2	77.4	16.1	6.5	Kebriaei P 2009
8	6 adults	40	2/4	5	0	1	83.3	0.0	16.7	Fang B 2007
9	12 children	7	10/2	7	5	0	58.0	42.0	0.0	Prasad VK 2010
10	2 adults	32	1/1	0	2	0	0.0	100	0.0	Lim JH 2010
11	3 adults	48	1/2	0	1	2	0	33.3	66.7	Arima N 2010
12	11 children	9	8/3	3	5	3	27.3	45.4	27.3	Lucchini G 2010
13	2 children	13.5	1/1	2	0	0	100	0	0	Fang B 2007
14	2 children	11.5	1/1	2	0	0	100	0	0	present study

	mean age range	27.0 0.5-67	65.2/34.8%							

Apart from the present study only those studies were included that reported on at least 2 individuals and that were available at MEDLINE^® ^(NCBI) until June 2011. CR = complete response; PR = partial response; NR = no response; ns = not specified. Children was defined as age < 18 years.

**Table 2:** Summary of patient outcome in clinical studies listed in Table 1

	# of patients		outcome (total)			outcome (%)		
patient category	total	%	CR	PR	NR	CR	PR	NR
children	61	33.3	35	15	11	57.4	24.6	18.0
adults	122	66.7	55	30	37	45.1	24.6	30.3

CR = complete response; PR = partial response; NR = no response. Children was defined as age < 18 years.

## Case presentations

Here we present two pediatric cases, which impressively demonstrate the beneficial effects of MSCs in treatment of steroid-refractory acute GvHD (compassionate use basis). For MSCs expansion protocols and release criteria please see Additional File 1.

### Case A

**Figure 1:**
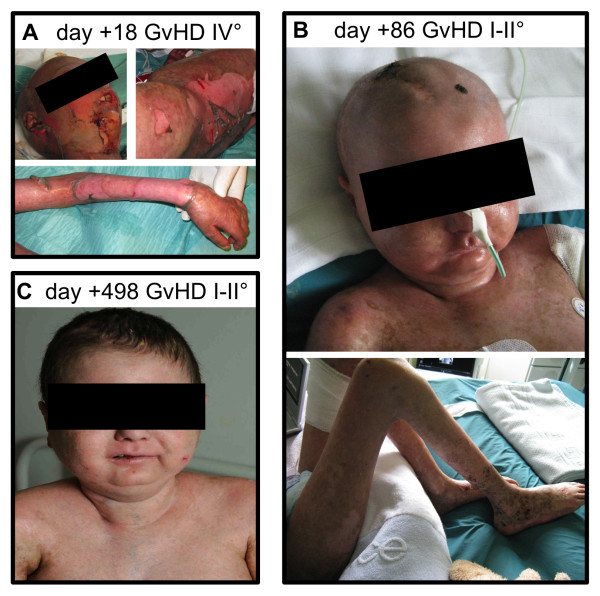
**Representative images of the skin of Case A demonstrating the course of cutaneous GvHD**: **A, **Images show severe acute GvHD (grade IV) of the face, the left lower back region and left forearm 18 days after haploidentical SCT (= 4 days before application of third-party MSCs). **B, **Images taken at day +86 after haploidentical SCT (= 60 days after transplantation of MSCs) show an intact skin with remaining manifestations of GvHD grade I-II. **C, **Image of the face and upper chest showing an intact skin (day +498 after haploidentical SCT, corresponding to day +482 after transplantation of MSCs). Written informed consent was obtained from the patient's legal guardian for the depiction of images that may identify individuals.

Our first patient is the only child of healthy non-consanguineous Caucasian parents. The boy was diagnosed with pre-B-ALL at the age of 3 4/12 years. Multimodal therapy was administered according to the ALL-BFM 2000 protocol in the high-risk group (risk factor: high minimal residual disease load before protocol M). Accordingly, he received an unrelated matched donor allogeneic SCT of a female donor. After SCT he developed acute GvHD of the skin, which continuously turned into extensive chronic GvHD of the skin including sclerodermiformal changes of the joints. He demonstrated persistent thrombocytopenia, one of the major risk factors indicative for poor prognosis in chronic GvHD [68]. The acute and chronic GvHD was treated with Ciclosporin A, glucocorticoids, Mycophenolate Mofetil, Psoralen and UV-A (PUVA) and extracorporal photopheresis (ECP). However, although ECP induced a significant improvement, extensive cicatrices and contractures of the skin and joints remained. At the age of 8 7/12 years (4 years after allogeneic SCT), he was again admitted to hospital due to progressive pancytopenia. Cytological analysis of bone marrow and peripheral blood showed leukemic blasts. Surprisingly, these blasts were not of lymphoid, but of myeloid origin and had a female karyotype (chimerism 100%, karyotype of the blasts 46;XX). Thus the diagnosis of a donor-derived AML (M2 according to FAB classification; NPM1b positive) was established. The patient was treated according to the protocol for relapsing AML [69], but only transient remission was achieved. Subsequently, the patient underwent haploidentical SCT (donor: mother) after conditioning with Fludarabin (3 × 50 mg/m^2^), Melphalan (70 mg/m^2^) and Thiotepa (10 mg/kg of recipient weight). In total 13.4 × 10^6 ^CD34^+ ^cells (CD3/CD19 depleted)/kg of recipient weight were transplanted without complications. For GvHD prophylaxis, we administered OKT-3 (0.1 mg/kg) and Methylprednisolone (2 mg/kg) from day -7 on, and added Mycophenolate Mofetil (20 mg/kg) on day +8. Engraftment took place on day +10. On day +12 after SCT, the patient developed acute dyspnea leading to acute respiratory distress syndrome (ARDS), followed by fulminant GvHD of the skin (grade IV) with bullous epidermiolysis of the entire epidermis (GvHD of the skin was proven by biopsy). Other organs were not involved. No improvement could be achieved with high-dose steroid therapy (Methylprednisolone 10 mg/kg) and addition of Cyclosporin A (6 mg/kg). The clinical condition of the patient deteriorated continuously. Steroid-refractory acute GvHD could not be controlled and we therefore decided to administer third-party MSCs (0.9 × 10^6 ^CD73^+^/CD105^+ ^cells/kg of recipient weight, obtained from an unrelated female donor). These were given intravenously at day +26 after allogeneic SCT as single infusion. No adverse effects and/or complications were observed during transplantation of the MSCs. No further infusion of MSCs was performed. Within the subsequent 4 weeks the skin recovered completely, without additional scars and contractures (Figure 1). Moreover, the clinical responsiveness upon conventional immunosuppressants improved and the applied dosages could be reduced successively without flaring of GvHD. To date (day +498 after allogeneic SCT), there is no evidence for leukemic relapse and only mild signs of an active chronic GvHD are present (e.g. reddish complexion of the skin, NIH classification grade I - II).

### Case B

Our second case is a 14 5/12-year-old girl suffering from an alveolar rhabdomyosarcoma of the left nasal cavity with cervical, mandibular and axillary metastases as well as affection of pelvic bone and bone marrow at time of diagnosis (stage IV according to NIH classification) [70]. Molecular analysis of the tumor cells revealed a *PAX3-FKHR *(paired box 3 - forkhead box O1) translocation that is usually associated with very poor outcome (3-year event-free survival < 10%) [71,72].

Induction chemotherapy was administered according to the CWS IV 2002 protocol in the high-risk arm, designed for the treatment of soft tissue sarcomas [73,74]. Additionally, a hyperfractionated radio-tomotherapy of the primary tumor region in the left rear nasal cavity, the paranasal sinuses and the cervical and axillary lymph nodes was conducted with a cumulative dose of 50 Gy (fractions of 2 Gy). Furthermore, two autologous SCTs were performed after conditioning with Melphalan/VP16 and Topotecan/Treosulfan, respectively, based on the Meta-EICESS protocol for multifocal Ewing tumors [1,75]. In addition, the patient underwent haploidentical SCT (4.91 × 10^6 ^CD34^+ ^cells (CD3/CD19 depleted)/kg of recipient weight) assuming the impact of a potential graft versus tumor effect (GvTE) [57,76]. GvHD prophylaxis was performed with OKT-3. Engraftment took place on day +15.

37 days later, the girl developed progressive diarrhea. The increasing frequency and volumes of gastrointestinal fluid loss culminated in up to 14.5 L/day at day +55, equivalent to acute GvHD grade IV of the gut that required hospitalization on intensive care unit. Liver and skin were not affected. GvHD was poorly responsive to the treatment with Methylprednisolone (5-10 mg/kg), Mycophenolate Mofetil (40 mg/kg), Cyclosporin A (according to blood level) and Etanercept 25 mg every two weeks. Immune-modulatory and regenerative properties of MSCs and reports on treatment of GvHD in the literature encouraged us to administer third-party MSCs (1.98 × 10^6 ^CD73^+^/CD105^+ ^cells/kg of recipient weight, obtained from an unrelated male donor). MSCs were transplanted as single infusion without complications or acute adverse effects. No further infusion of MSCs was performed. Within 5 days after intravenous application of MSCs, the frequency of diarrhea decreased to approximately one half. At day +16 after treatment with MSCs, the patient was able to return to outpatient care without signs of active GvHD and evidence of residual tumor masses. Unfortunately, on routine follow-up screening 18 months after allogeneic SCT, the patient was found to have extensive relapse with metastasis (proven by biopsy) and she is currently receiving salvage therapy with donor-lymphocyte infusions and hyperthermia.

## Meta-analysis

As discussed above, a few reports on the efficacy of MSCs in treatment of GvHD in children indicate that children tend to respond better on treatment with MSCs than adults. To prove if this trend holds true we performed a meta-analysis of available clinical reports and trials concerning the treatment of steroid-refractory GvHD with MSCs. Apart from the present study only those studies were included that reported on at least 2 individuals and that were published in a peer-reviewed journal available at MEDLINE^® ^(NCBI) until June 2011. Studies reporting on co-transplanted MSCs to prevent GvHD were not considered. A total of 13 relevant original articles was identified (reporting in summary on 183 patients; 122 adults, 61 children) and pertinent data were analyzed using OpenEpi 2.3 software http://www.openepi.com/OE2.3. As seen from Table 1 and Table 2 most patients did respond to the treatment of steroid-refractory GvHD with MSCs (overall response-rate 73.8%). Furthermore, our meta-analysis confirms that children indeed responded better than adults (complete response 57.4% vs. 45.1%, respectively). Comparing the rates of responders (complete and partial response) vs. non-responders in adults and children, we found that 82.0% of the children did respond to treatment of GvHD with MSCs compared to 69.7% of the adults (risk difference: 12.3%; odds ratio 1.972, 95% CI 0.94 - 4.37; *P *= 0.037, Mid-P exact test).

## Discussion and conclusions

Severe acute and chronic GvHD is a life-threatening complication after allogeneic hematopoietic SCT [4,5]. Despite major adverse effects, steroids are still essential in first-line therapy of acute and chronic GvHD [2,3]. The response-rates, however, are as low as 30-50% and the outcome for steroid-refractory acute GvHD is poor [2,3]. Furthermore, prolonged and extensive use of pharmacological immunosuppressants is associated with high risk of viral reactivation and fungal infections [77,78]. To date, several non-pharmacological treatment options like extracorporal photopheresis are employed to treat acute GvHD and to reduce dosages of conventional immunosuppressants [5,79]. Eventually, also third-party MSCs might be an additional non-pharmacological treatment option to reduce immunosuppressants, although this supposition clearly has to be tested in future studies.

As demonstrated by the presented cases and our meta-analysis, third-party MSCs seem to be an attractive therapeutic strategy in steroid-refractory acute GvHD after allogeneic SCT also in children. The reason for the seemingly better response-rates in children than in adults still needs to be elucidated. Although, interpretation of this observation is difficult and perhaps preliminary in nature, it is tempting to speculate that specific stromal factors of children facilitate the engraftment of MSCs and ultimately function of MSCs compared to adults.

To gain more functional insights in these phenomena, it has been recently suggested to label transplanted MSCs for more efficient tracking and imaging in patients in order to monitor their kinetics of expansion and location [34]. Moreover, older recipient age has been identified as an important risk factor for poor outcome in acute and chronic GvHD [80-82], which possibly contributes to the difference in outcome between adult and pediatric patients as seen in our meta-analysis. Certainly, further experimental work and clinical studies are required to address this issue.

In line with the assumption that suppression of GvHD can result in a decrease of GvTE, some studies have shown that therapeutic prevention of acute GvHD may result in increased relapse rates [83,84], while other studies indicate that co-transplanted MSCs might not decrease GvTE [85]. Although it is unclear if treatment of an already established acute GvHD by third-party MSCs might increase relapse rates, it is noteworthy that as yet there has been no evidence for MSCs-associated tumorigenesis in clinical trials, as well as that there appears to be no increase in rates of DNA viral infections or post-transplantation lympho-proliferative disease (PTLD) [33-35].

Facing the dramatic course of acute GvHD in our cases, we decided to administer MSCs as an ultimate salvage therapy on a compassionate use basis that indeed proved to control symptoms of GvHD. The successful treatment of life-threatening GvHD in our patients and the high overall response-rates seen in our meta-analysis leads us to the conclusion that MSCs should be considered as a feasible treatment option for adults and children with severe steroid-refractory GvHD.

## Consent

Written informed consent was obtained from the patients and/or their legal guardians for publication of their medical history. Copies of the written consents are available for review by the Editor-in-Chief of this journal.

## Abbreviations

ALL: acute lymphoblastic leukemia; ALL-BFM: Akute Lymphatische Leukaemie - Berlin-Frankfurt-Muenster; AML: acute myeloid leukemia; ARDS: acute respiratory distress syndrome; COL7A1: collagen, type VII, alpha 1; CWS: Cooperative Weichteilsarkom Studie; ECP: extracorporal photopheresis; FAB: French-American-British; GvHD: graft versus host disease; GvTE: graft versus tumor effect; HLA: human leukocyte antigen; HSCs: hematopoietic stem cells; IFNγ: interferon γ; MSCs: mesenchymal stromal cells; NPM1b: nucleophosmin 1b; *PAX3-FKHR*: paired box 3-forkhead box O1 translocation; PTLD: post-transplantation lymphoproliferative disease; PUVA: Psoralen and UV-A; SCT: stem cell transplantation; TNFα: tumor necrosis factor α; T-regs: regulatory T-cells.

## Competing interests

The authors declare that they have no competing interests.

## Authors' contributions

CW, TG, HJ, SK, IM, and IvL drafted and wrote the paper. CW and TG conducted the meta-analysis. SK, ZK, MD, UK, PB, and IM were involved in MSCs preparation. All authors were involved in literature review and patient care and approved the final manuscript.

## Supplementary Material

Additional file 1 **MSCs expansion and release criteria**. This file contains a detailed description of the MSCs expansion and release criteria for Case A and Case B. Click here for file
