# Supplementary material for: Mesenchymal stromal cells for treatment of steroid-refractory GvHD: a review of the literature and two pediatric cases
Source: Int Arch Med. 2011 Aug 15;4:27. doi: 10.1186/1755-7682-4-27 (PMC3169455; doi:10.1186/1755-7682-4-27)
Supplement: Additional file 1 — MSCs expansion and release criteria. This file contains a detailed description of the MSCs expansion and release criteria for Case A and Case B. [file 1755-7682-4-27-S1.DOC]

**ADDITIONAL FILE 1**

**MSCs expansion and release criteria:**

Due to limited availability but in parallel urgent requirement of MSCs for treatment of our patients, we obtained MSCs from two different institutions (University of Tuebingen and University of Frankfurt am Main, respectively). Standardized expansion protocols, each with slight modifications, and release criteria were as follows:

*MSCs expansion and release criteria for Case A:* 20ml heparinized bone marrow were harvested by iliac crest puncture from a healthy 45-year-old female donor (previously tested for viral infections) using a protocol approved by the University of Tuebingen Institutional Review Board. All cell culture steps were performed in a GMP facility. Red blood cells were removed by ammonium chloride lysis for 10 min at room temperature. Lysis buffer consisted of 8.29g ammonium chloride, 1g potassium hydrogencarbonate, 0.037g Na_2_EDTA/H_2_O, added to 1000ml ddH_2_O and filter sterilized. The remaining cells were washed once in PBS. Cells were resuspended in 50ml of low glucose DMEM (Cambrex, Verviers, Belgium) supplemented with 1mM Glutamine (Cambrex), 5% (v/v) human fresh frozen plasma (FFP), 5% platelet lysate (from single donor platelet concentrate at 10^8^/μl, University of Tuebingen blood donor center) and 80IU/ml heparin sulphate. Cells were incubated at 37°C under 10% CO_2_ in a T175 tissue culture flask with vented cap. After 48h non-adherent cells were removed. At 80% confluency cells were detached with recombinant trypsin, washed and re-plated in two T175 tissue culture flasks, then 10 and after 2 weeks finally in 40 flasks (three passages in total). MSCs were harvested, washed, resuspended in 50ml physiological NaCl solution containing 0.1% human albumin (Octapharma, Langefeld, Germany), passed through a 70 μm cell strainer (Corning Inc., Corning, NY) and transfused over a period of 15 min. Release criteria included culture for less than 6 weeks, viability >75%, absence of T-cells, lack of CD45 and expression of CD73, CD90 and CD105 by more than 90% of the cells as assessed by flow cytometry. Sterility tests were performed at the start of the culture and during the last passage 1 week before administration.

*MSCs expansion and release criteria for Case B:* 30ml of bone marrow aspirate were obtained from the posterior iliac crest of a healthy 17-year-old male donor (previously tested for viral infections) under whole anesthesia using a protocol approved by the University of Frankfurt am Main Institutional Review Board. All cell culture steps were performed in a GMP facility. The bone marrow aspirate was diluted 1:2 in PBS and after that two volumes of the samples were layered over 1 volume ficoll (density: 1.077 g/ml) (Biochrom, Berlin, Germany) in a 50ml conical tube and centrifuged at 700 x g for 30 min without brake. Mononuclear cells were then collected from the interface, washed twice with PBS and centrifuged at 400 x g for 10 min. The cell pellet was resuspended in low-glucose DMEM with GlutaMAX™ I (Gibco/Invitrogen, Darmstadt, Germany) supplemented with 10% of MSCs-qualified FBS (Invitrogen) and plated in T175 tissue culture flasks with vented caps at a concentration of 1.7x10^5^ cells/cm^2^. The cells were cultured in an incubator with 5% CO_2_ and 95% humidity at 37°C. After 72h non-adherent cells were removed, whereas the adherent cell fraction was further cultured in fresh culture medium. Culture medium was changed every 4 days and once the MSCs reached a 70-80% confluence, they were detached with recombinant trypsin (TrypLE™ Select, Invitrogen), washed and further re-plated in new T175 tissue culture flasks. After 3 passages (P3) the MSCs were frozen in DMEM supplemented with 30% FBS and 10% DMSO. Before transplantation the MSCs were thawed and washed 3 times with CliniMACs buffer (Miltenyi Biotec, Bergisch-Gladbach, Germany) supplemented with 0.5% human serum albumin (HSA) (Baxter, Heidelberg, Germany). After the last wash step the MSC pellets were collected and resuspended in 100 ml CliniMACS buffer supplemented with 0.5% human albumin and administered to the patient. Release criteria included sterility tests performed at the start of the culture and at the end of passage 3 before MSCs were cryoconserved, viability >75%, absence of T-cells, lack of CD45 and expression of CD73, CD90 and CD105 by more than 90% of the cells as assessed by flow cytometry.
